# A comprehensive approach to identify approved drugs and treatments for repositioning as therapies for systemic lupus erythematosus

**DOI:** 10.1186/ar4667

**Published:** 2014-09-18

**Authors:** Amrie C Grammer, Matthew Ryals, Peter E Lipsky

**Affiliations:** 1 AMPEL BioSolutions, Charlottesville, VA, USA

## Background

Development of new systemic lupus erythematosus (SLE) treatments has been slow. To accelerate the pace, an evidence-based approach was developed to find new lupus therapies amongst 6,800 compounds FDA approved for human use.

## Methods

The Lupus Treatment List (LRxL) was constructed with intense input from the entire lupus community, including patients, and was used to prioritize therapies to be tested in small focused, biomarker-rich clinical trials (SLE Treatment Acceleration Trials (STAT)). All drugs widely used for lupus or known to be in development for lupus by Pharma/Biotech were excluded. Details of the project can be viewed online [1]. A novel evidence-based composite scoring system was developed to rank the identified drugs/therapies numerically by scientific rationale, experience in lupus mice/human cells, previous clinical experience in autoimmunity, drug properties and adverse event profile.

## Results

Of the 157 therapies initially screened, more than 25 have an appropriate set of characteristics to consider for testing in clinical trials in lupus, including drugs targeting cellular metabolism, kinases, the immune system, HDACs, complement as well as cellular therapies and nondrug interventions.

## Conclusions

This approach has not only identified unique candidates that could be useful in SLE and possibly other autoimmune/inflammatory conditions, but has also yielded a rigorous evidence-based process by which therapies can be usefully rated for possible clinical application to treat these conditions, thereby mitigating risk in drug development.
